# Calcium hydroxide nanoparticles induce apoptotic cell death in human pancreatic cancer cells through over ROS-driven genomic instability and mitochondrial dysfunction

**DOI:** 10.1038/s41598-025-26135-5

**Published:** 2025-11-18

**Authors:** Hanan R. H. Mohamed, Hagar Magdy, Yusuf Elberry, Maryam Ismail, Maivel Michael, Nourhan Eltayeb, Gehan Safwat, Ayman Diab

**Affiliations:** 1 Department of Zoology, Faculty of Science, Cairo University, Giza, Egypt; 2 Faculty of Biotechnology, October University for Modern Sciences and Arts (MSA), 6th of October City, Egypt

**Keywords:** Ca(OH)_2_ NPs, PANC-1 cells, DNA stability, ROS generation, Mitochondrial apoptosis, Biochemistry, Cancer, Cell biology, Drug discovery

## Abstract

The aggressive nature of pancreatic cancer, coupled with the limitations of current treatment options, underscores the urgent need for more effective and targeted therapies. Nanoparticle-based approaches offer promising alternatives, with calcium hydroxide nanoparticles (Ca(OH)_2_ NPs) emerging as a potential candidate due to their biocompatibility, high alkalinity, and ability to modify the tumor microenvironment. However, their therapeutic potential against pancreatic cancer remains largely unexplored. This study thus estimated the effects of Ca(OH)_2_ NPs on the viability of normal oral epithelial cells (OECs) and pancreatic cancer PANC-1 cells, moreover, the impact of Ca(OH)_2_ NPs on genomic DNA and mitochondrial membrane integrity, reactive oxygen species (ROS) generation, and apoptosis induction in PANC-1 cells was assessed. Sulforhodamine B cytotoxicity assay demonstrated a strong, targeted concentration-dependent cytotoxic effect of Ca(OH)_2_ NPs on PANC-1 cells following exposure to five different concentrations (0.01, 1, 10, 100, and 1000 µg/ml) for 72 h, with an IC50 value of 152.40 µg/ml. In contrast, minimal cytotoxicity was observed in normal OECs, which had an IC50 value of 481.66 µg /ml. The calculated selectivity index of 3.16 further confirmed the preferential cytotoxicity of Ca(OH)_2_ NPs towards PANC-1 cells. Moreover, exposure of PANC-1 cells to the IC50 concentration of Ca(OH)_2_ NPs (152.40 µg/ml) led to excessive ROS generation, marked genomic instability, and loss of mitochondrial membrane integrity. These effects were accompanied by dysregulation of key apoptotic genes, including upregulation of p53 and mitochondrial ND3, along with downregulation of the anti-apoptotic Bcl-2 gene, ultimately inducing mitochondrial apoptosis in PANC-1 cells. Ca(OH)_2_ NPs exhibit potent, selective cytotoxicity against PANC-1 cells while exerting minimal toxicity on normal OECs. Their mechanism of action appears to involve excessive ROS generation, leading to severe genomic DNA and mitochondrial damage, ultimately triggering apoptosis in pancreatic cancer cells. These findings highlight the potential of Ca(OH)_2_ NPs as a novel therapeutic agent for pancreatic cancer. However, further in vitro and in vivo studies are warranted to fully explore their clinical applicability and underlying molecular mechanisms in pancreatic cancer treatment.

## Introduction

Cancer remains a major global health burden, necessitating the continuous search for novel therapeutic agents with selective cytotoxic effects on cancer cells while sparing normal cells^1^. Among the most aggressive malignancies, pancreatic cancer has one of the lowest survival rates, with a five-year survival below 10% due to late diagnosis, rapid progression, and limited treatment options. Pancreatic ductal adenocarcinoma (PDAC), the most common form, is highly resistant to conventional therapies, making it a significant challenge in oncology^2–4^.

Current treatment strategies for pancreatic cancer primarily include surgical resection, chemotherapy, and radiation therapy. However, chemotherapy, particularly gemcitabine-based regimens, remains the cornerstone of treatment for advanced pancreatic cancer despite its limited efficacy and severe toxic side effects^5^. The major challenges associated with conventional chemotherapy include systemic toxicity, non-specific drug distribution, and the development of chemoresistance, which significantly reduces treatment effectiveness^6,7^.

In response to these limitations, nanotechnology-based therapies have emerged as a promising alternative, offering enhanced drug delivery, improved tumor targeting, and reduced systemic side effects^8,9^. Among these, calcium hydroxide nanoparticles (Ca (OH)_2_ NPs) have gained attention due to their unique properties, including high alkalinity, selective cytotoxicity, and potential to induce oxidative stress in cancer cells while exhibiting minimal toxicity to normal cells^10,11^.

The findings of Mohamed et al.^11^, study revealed the selective genotoxicity of Ca(OH)_2_ NPs towards hepatic Hep-G2 cancer cells through excessive reactive oxygen species (ROS) generation that caused dramatic DNA damage and alterations of apoptotic and anti apoptotic gene expression triggering apoptosis of Hep-G2 cells without any changes in genomic stability and generation of ROS in normal human skin cells (HSF), highlighting their ability to selectively induce apoptosis in cancer cells, making them a promising alternative to traditional chemotherapy.

While nanotechnology has been widely explored in cancer therapy, the potential of Ca(OH)_2_ NPs remains largely unexamined, particularly in pancreatic cancer. Unlike conventional chemotherapy, which is associated with severe toxicity and drug resistance, Ca(OH)₂ NPs offer a unique mechanism involving reactive oxygen species (ROS) generation, mitochondrial dysfunction, and selective apoptosis induction in cancer cells^11,12^.

By evaluating their selective cytotoxicity and genotoxic effects, this study provides new insights into the potential application of Ca(OH)_2_ NPs as a targeted nanotherapeutic agent, offering a safer and more effective alternative to traditional treatments. Therefore, this study was thus conducted to explore their cytotoxic effects of Ca(OH)_2_ NPs on normal human oral epithelial cells (OEC) and pancreatic PANC-1 cancer cells. Moreover, the possibility of excessive ROS generation, loss of mitochondrial membrane potential, genomic instability and apoptosis induction by Ca(OH)_2_ NPs were estimated in human pancreatic PANC-1 cells, providing new insights into their applicability as a targeted nanotherapeutic agent for pancreatic cancer.

## Materials and methods

### Preparation and characterization of Ca(OH)_2_ NPs

Ca(OH)_2_ NPs in white powder form with an average particle size of < 100 nm) were obtained from NanoTech Company, Cairo, Egypt. Before each experiment, Ca(OH)₂ NPs were freshly dispersed in dimethyl sulfoxide (DMSO, ) to prepare the required stock solution (1 mg/mL) and subsequently diluted to working concentrations (0.01–1000 µg/mL). The suspensions were ultrasonicated (40 kHz, 30 min) using a digital ultrasonic bath (Sonica, Italy) to ensure uniform dispersion and minimize aggregation.

**Fig. 1:**
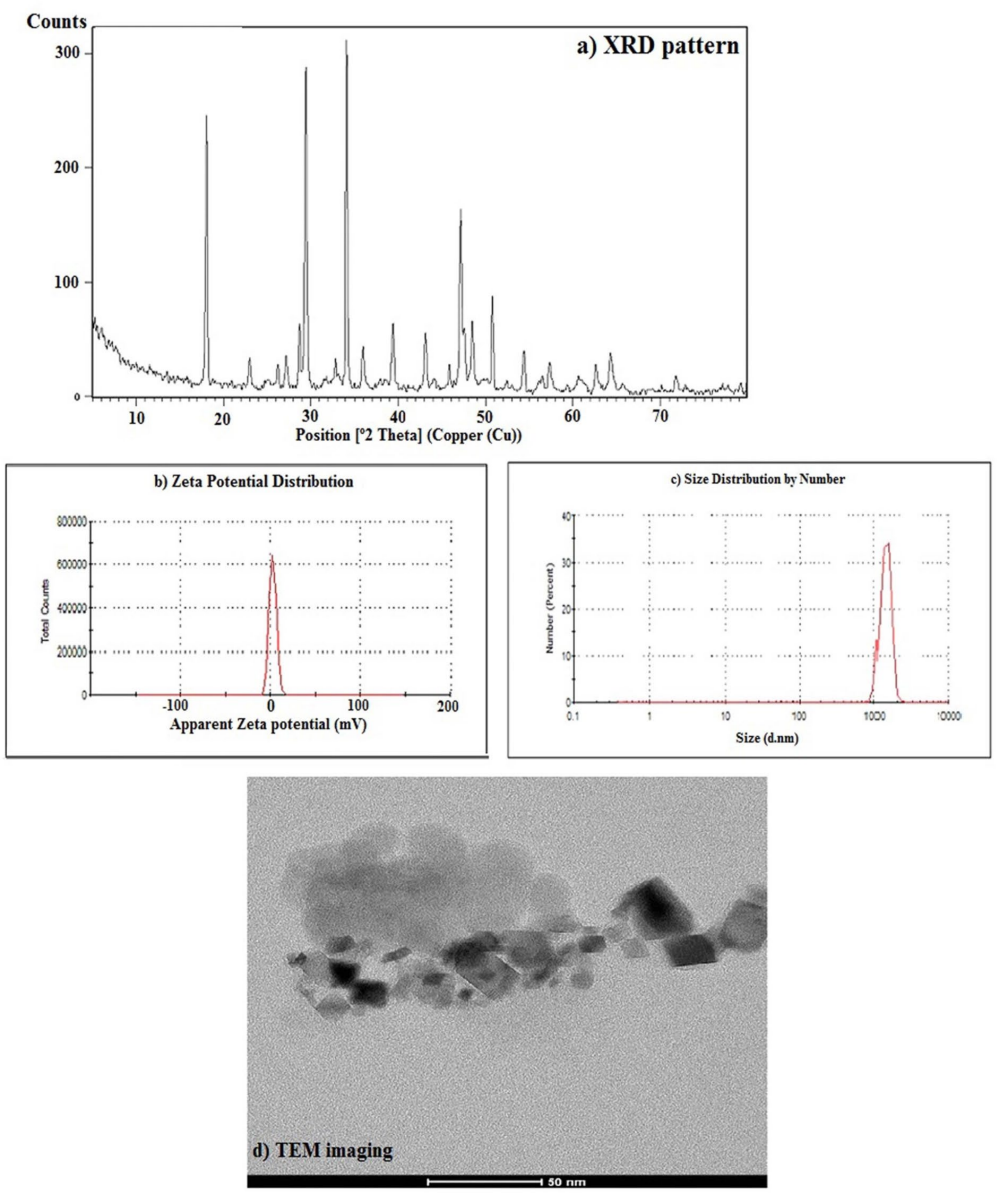
Characterization profile of Ca(OH)_2_ NPs showing (**a**) X-ray diffraction (XRD) pattern; (**b**) Zeta Potential distribution; (**c**) Particle Size Distribution and (**d**) Transmission electron microscopy (TEM) imaging (Mohamed et al., 2025).

The physicochemical characterization of the Ca(OH)₂ NPs used in this study was previously performed and published by Mohamed et al.^11^. X-ray diffraction (XRD) analysis confirmed high crystallite purity with distinctive diffraction peaks at 2θ = 18°, 29°, and 34°, characteristic of Ca(OH)₂. Dynamic light scattering (DLS) analysis revealed a zeta potential of + 2.45 mV and a polydispersity index (PDI) of 0.416, indicating a tendency for aggregation that necessitated ultrasonication prior to use. Transmission electron microscopy (TEM) further demonstrated well-dispersed cubic to spherical nanoparticles with an average particle size of 12.4 ± 2.4 nm (Fig. 1).

### Pancreatic PANC-1 and oral epithelial OEC cell culture

Human pancreatic cancer (PANC-1) and normal oral epithelial (OEC) cell lines were obtained from Nawah Scientific Inc. (Mokatam, Cairo, Egypt). Cells were maintained separately in Dulbecco’s Modified Eagle Medium (DMEM) supplemented with 10% heat-inactivated fetal bovine serum (FBS), 100 U/mL penicillin, and 100 µg/mL streptomycin. Cultures were incubated at 37 °C in a humidified atmosphere containing 5% CO₂ and sub-cultured every 2–3 days to maintain exponential growth.

### Cytotoxicity assessment using SRB assay

The cytotoxic potential of Ca(OH)₂ NPs was evaluated using the Sulphorhodamine B (SRB) assay^13,14^. OEC and PANC-1 cells were seeded at a density of 1 × 10⁴ cells/well into 96-well plates and incubated for 24 h to allow cell attachment. Cells were then treated with five concentrations of Ca(OH)₂ NPs (0.01, 1, 10, 100, and 1000 µg/mL) for 72 h. Untreated and vehicle-treated cells served as negative controls. After incubation, cells were fixed with 10% trichloroacetic acid (TCA), washed with distilled water, and stained with 0.4% SRB solution for 10 min in the dark. Excess dye was removed using 1% acetic acid, and the plates were air-dried overnight. Protein-bound dye was solubilized in 10 mM Tris buffer (pH 10.5), and absorbance was measured at 540 nm using a BMG LABTECH FLUOstar Omega microplate reader (Ortenberg, Germany). The IC50 values were calculated using GraphPad Prism (v9.0) from three independent experiments (each in triplicate). The selectivity index (SI) was computed by dividing the IC50 value of normal OEC cells by the IC50 value of pancreatic PANC-1 cancer cells.

### Treatment schedule

PANC-1 cancer cells were cultured in T25 flasks, divided into untreated controls and Ca(OH)₂ NP-treated cells. The treated group received Ca(OH)₂ NPs at the IC50 concentration (152.40 µg/mL) for 72 h. Controls received culture medium containing < 0.1% DMSO. After treatment, cells were harvested by trypsinization, centrifuged, and washed twice with ice-cold phosphate-buffered saline (PBS, pH 7.4). Cell pellets were stored at − 80 °C for subsequent molecular and biochemical analyses. All experiments were conducted in triplicate biological replicates, each performed in technical triplicate.

### Measurement of ROS generation level

ROS generation in treated and untreated PANC-1 cells was quantified using 2’,7’-dichlorofluorescin diacetate (DCFH-DA)^15^. Cells were incubated with 20 µM DCFH-DA for 30 min at room temperature in the dark. The dye was oxidized by intracellular ROS to form fluorescent dichlorofluorescein (DCF). Cells were examined under an epifluorescence microscope (200× magnification), and fluorescence intensity was quantified using Fiji ImageJ software, where increased green fluorescence indicated elevated ROS levels.

### Assessment of genomic DNA using alkaline comet assay

Induction of DNA damage by Ca(OH)_2_ NPs in PANC−1 cancer cells was assessed using the alkaline comet assay^16,17^. After 72 h of exposure to Ca(OH)₂ NPs at the IC50 concentration, treated and control PANC-1 cancer cells were embedded in 0.5% low-melting agarose on pre-coated slides, lysed in cold buffer (with freshly added DMSO and Triton X-100) at 4 °C for 24 h, and subjected to electrophoresis in alkaline buffer (pH > 12) at 25 V, 300 mA for 30 min. Slides were neutralized, stained with ethidium bromide, and imaged under a fluorescence microscope. DNA damage parameters; tail length, % DNA in tail, and tail moment, were analyzed using CometScore™ software and reported as mean ± SD.

### Evaluation of the mitochondrial membrane potential

Mitochondrial membrane potential was determined using Rhodamine-123 (Rh-123) fluorescent dye^18^ following PANC-2 cell exposure to the IC50 concentration of Ca(OH)_2_ NPs for 72 h. Equal volumes of cell suspension and Rh-123 solution (10 µg/mL) were mixed and incubated at 37 °C in the dark for 1 h. After washing with PBS, cells were smeared on clean slides and observed under an epifluorescence microscope (200×). The fluorescence intensity was quantified using Fiji ImageJ, where a decrease in fluorescence indicated loss of mitochondrial membrane integrity.

### Flow cytometric analysis of apoptosis in PANC-1 cells

Apoptosis induction in untreated and Ca(OH)_2_ NPs-treated PANC−1 cells was quantified using Annexin V-FITC/Propidium Iodide (PI) dual staining (Abcam, UK) following the manufacturer’s protocol. After 72 h of treatment, cells were collected, washed with cold PBS, and incubated with Annexin V-FITC and PI for 30 min in the dark at room temperature. Samples were analyzed using an ACEA Novocyte flow cytometer (ACEA Biosciences, USA). FITC and PI emissions were detected in FL1 (488/530 nm) and FL2 (535/617 nm) channels, respectively. A minimum of 12,000 events per sample were recorded, and apoptotic populations were quantified using NovoExpress software.

### Quantification of *p53*,* ND3* and *Bcl2* gene expression in PANC-1 cells

Expression level of apoptotic-*p53*, mitochondrial *ND3*, and *Bcl-2* genes were quantified by quantitative real-time polymerase chain reaction (qRT-PCR). Total RNA was extracted using the GeneJET RNA Purification Kit (Thermo Fisher Scientific, USA) and reverse-transcribed into cDNA using the High-Capacity cDNA Reverse Transcription Kit (Applied Biosystems, USA). Amplification was performed using SYBR Green PCR Master Mix on a StepOnePlus™ Real-Time PCR System (Applied Biosystems). Primer sequences are listed in Table 1^19–21^. The housekeeping gene GAPDH served as the internal control. Relative gene expression levels were calculated using the 2⁻ΔΔCt method and expressed as mean ± SD from three independent experiments.

**Table 1:** Sequences of primers used in qRT-PCR.

Gene	Strand	Primer’s sequences
GAPDH	Forward	5ʹ-GAAGGTGAAGGTCGGAGTCA-3ʹ
	Reverse	5ʹ-GAAGATGGTGATGGGATTTC-3ʹ
ND3	Forward	5ʹ-CGCCGCCTGATACTGGCAT-3ʹ
	Reverse	5ʹ-CTAGTATTCCTAGAAGTGAG-3ʹ
BCL-2	Forward	5ʹ-TCCGATCAGGAAGGCTAGAGT-3ʹ
	Reverse	5ʹ-TCGGTCTCCTAAAAGCAGGC-3ʹ
P53	Forward	5ʹ-CAGCCAAGTCTGTGACTTGCACGTAC-3ʹ
	Reverse	5ʹ-CTATGTCGAAAAGTGTTTCTGTCATC-3ʹ

### Statistical analysis

The results from the alkaline comet assay and qRT-PCR were analyzed using the Statistical Package for the Social Sciences (SPSS) and are presented as mean ± standard deviation (SD). An unpaired Student’s t-test was performed to compare treated and untreated PANC-1 cells at a probability value of *p* < 0.05.

## Results

### Ca(OH)_2_ NPs exhibited selective strong cytotoxicity against PANC-1 cells

**Fig. 2:**
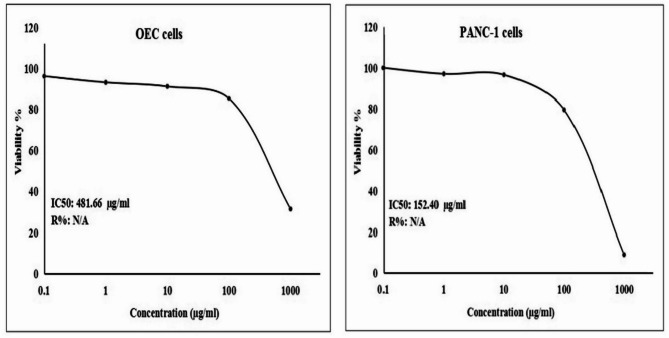
Viability of human normal OEC and pancreatic PANC-1 cells after 72 h of exposure to Ca(OH)_2_ NPs at five concentrations (0.1, 1, 10, 100, and 1000 µg/ml), measured using the SRB assay.

The findings of the SRB cytotoxicity assay confirmed the potent and selective effect of Ca(OH)_2_ NPs on pancreatic PANC-1 cancer cells, while showing minimal cytotoxicity toward normal OEC cells. As displayed in Fig. 2, this targeted cytotoxicity was evident through a significant concentration-dependent reduction in PANC-1 cell viability after 72 h of exposure to varying concentrations of Ca(OH)_2_ NPs (0.1, 1, 10, 100, and 1000 µg/mL), with an IC50 value of 152.40 µg/mL. In contrast, normal OEC cells exhibited only a slight decrease in viability under the same conditions, with an IC50 value of 481.66 µg/mL. The calculated selectivity index of 3.16 further confirmed the strong preferential cytotoxicity of Ca(OH)_2_ NPs toward PANC-1 cells. Consequently, the following molecular studies were conducted on pancreatic PANC-1 cancer cells to further explore the targeted cytotoxic potential of Ca(OH)_2_ NPs.

### Ca(OH)_2_ NPs induce excessive ROS generation in PANC-1 cells

**Fig. 3:**
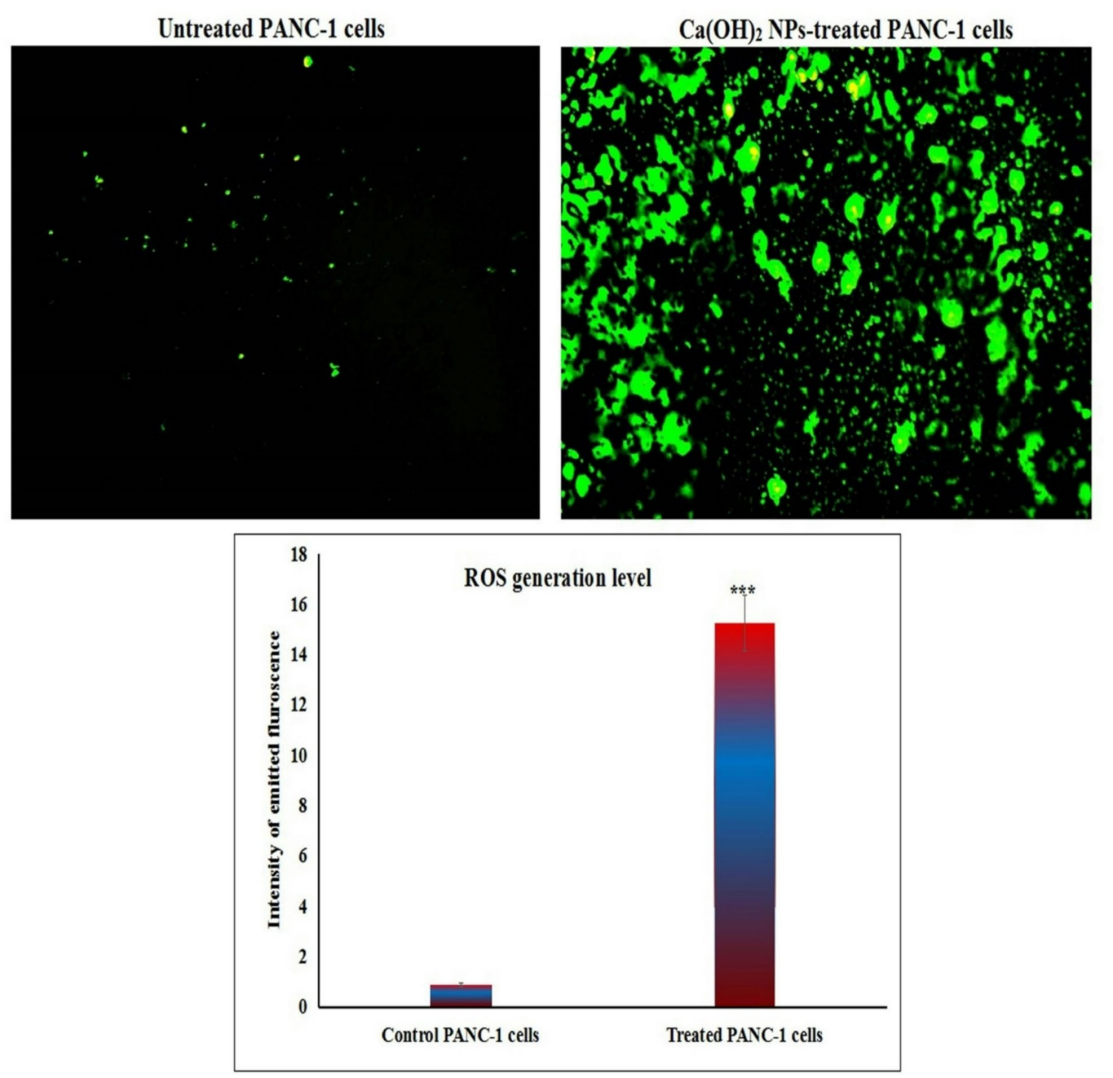
Generation of ROS within the untreated and treated pancreatic PANC-1 cancer cells with the IC50 concentration (152.40 µg/ml) of Ca(OH)_2_ NPs for 72 h. Magnification 200x.

Screening and analysis of DCFH-DA-stained PANC-1 cells demonstrated a significant (*p* < 0.001) overproduction of reactive oxygen species (ROS) following exposure to Ca(OH)_2_ NPs at the IC50 concentration (152.40 µg/mL) for 72 h. This was evidenced by a substantial increase in fluorescence intensity in Ca(OH)_2_ NPs-treated PANC-1 cells compared to untreated controls (Fig. 3).

### Ca(OH)_2_ NPs induce severe genomic DNA damage in PANC-1 cells

**Fig. 4:**
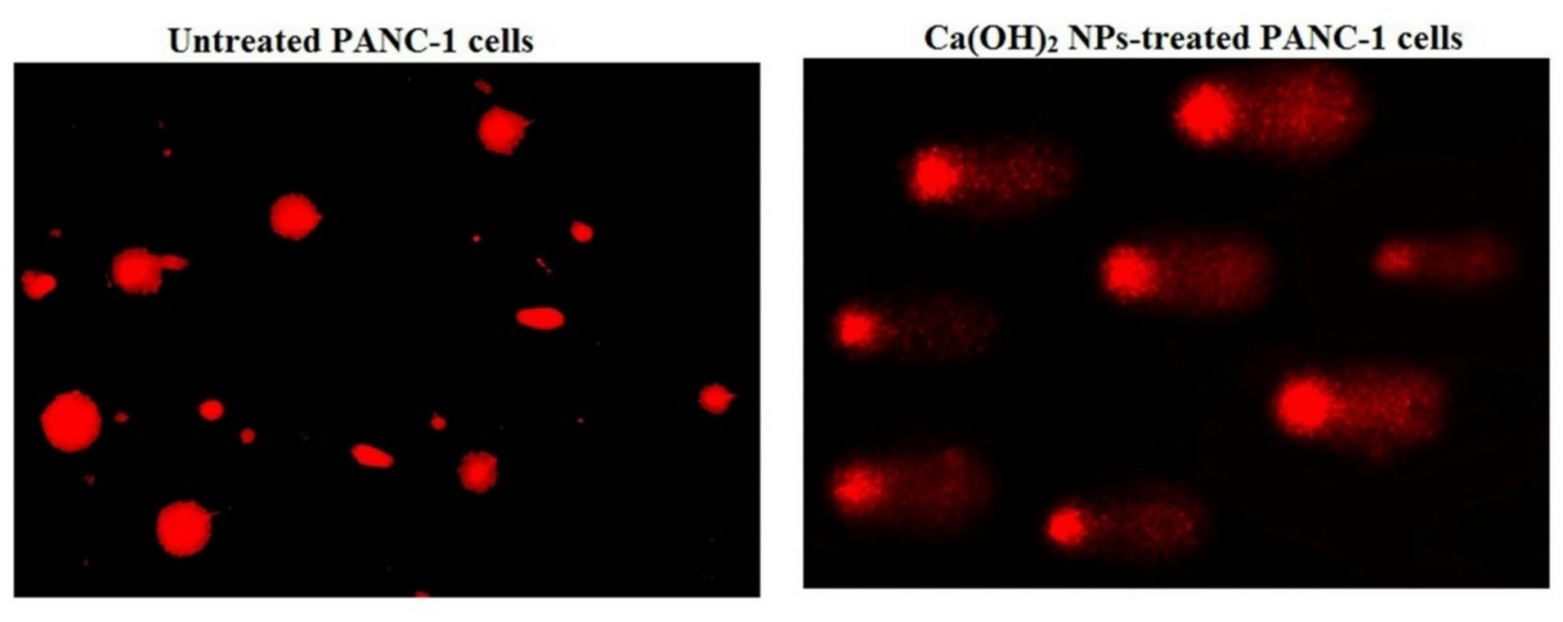
Examples for the scored Comet nuclei with intact DNA in (**a**) untreated PANC-1 cells and with damaged DNA in (**b**) PANC-1 cells treated with the IC50 concentration (152.40 µg/ml) of Ca(OH)_2_ NPs for 72 h. Magnification 200x.

The results of alkaline Comet assay displayed in Table 2; Fig. 4 demonstrated significant DNA damage in PANC-1 cancer cells following 72 h of treatment with Ca(OH)_2_ NPs. This was evidenced by statistically significant increases in tail length (*p* < 0.01), %DNA in tail (*p* < 0.01), and tail moment (*p* < 0.001) in Ca(OH)_2_ NPs-treated PANC-1 cells compared to untreated controls. Representative images of Comet nuclei with intact and damaged DNA are presented in Fig. 4.

**Table 2:** Genomic DNA damage induction in human pancreatic cancer (PANC-1) cells following exposure to Ca(OH)_2_ NPs at the IC50 concentration (152.40 µg/ml) for 72 h.

Cells	Treatment (µg/ml)	Tail length (px)	%DNA in tail	Tail moment
PANC-1 cells	Ca(OH)_2_ NPs (0.00)	4.20 ± 0.55	16.65 ± 1.82	0.70 ± 0.15
	Ca(OH)_2_ NPs (152.40)	14.35 ± 1.94^**^	31.90 ± 1.95^**^	4.37 ± 0.50^***^

Results are expressed as mean ± SD.

**, ***: Indicates statistical significant difference from the compared untreated control cells at *p* < 0.01 and *p* < 0.001, respectively, using *independent student t-test*.

### Ca(OH)_2_ NPs cause a dramatic loss of mitochondrial integrity in PANC-1 cells

**Fig. 5:**
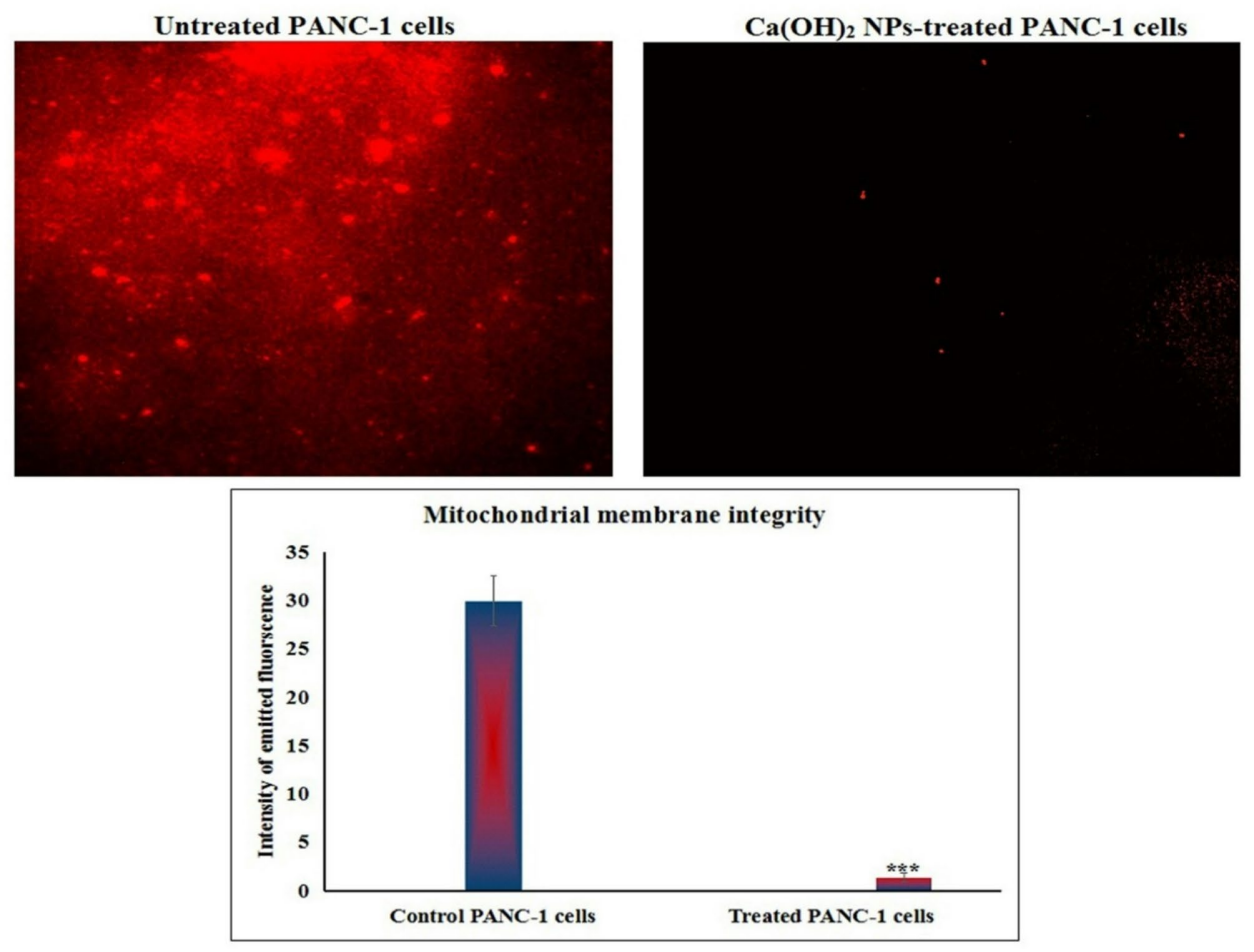
Dramatic loss of mitochondrial membrane integrity in the untreated and treated pancreatic PANC-1 cancer cells with the IC50 concentration (152.40 µg/ml) of Ca(OH)_2_ NPs for 72 h. Magnification 200x.

Staining of PANC-1 cancer cells with the fluorescent dye Rhodamine-123 revealed that exposure to the IC50 concentration (152.40 µg/mL) of Ca(OH)_2_ NPs for 72 h led to a substantial loss of mitochondrial membrane integrity (Fig. 5). This damage was evident from a significant (*p* < 0.001) reduction in the fluorescence intensity emitted by Ca(OH)_2_ NPs-treated PANC-1 cells compared to untreated control cells, indicating severe mitochondrial dysfunction (Fig. 5).

### Ca(OH)_2_ NPs trigger apoptosis and necrosis in PANC-1 cells

**Fig. 6:**
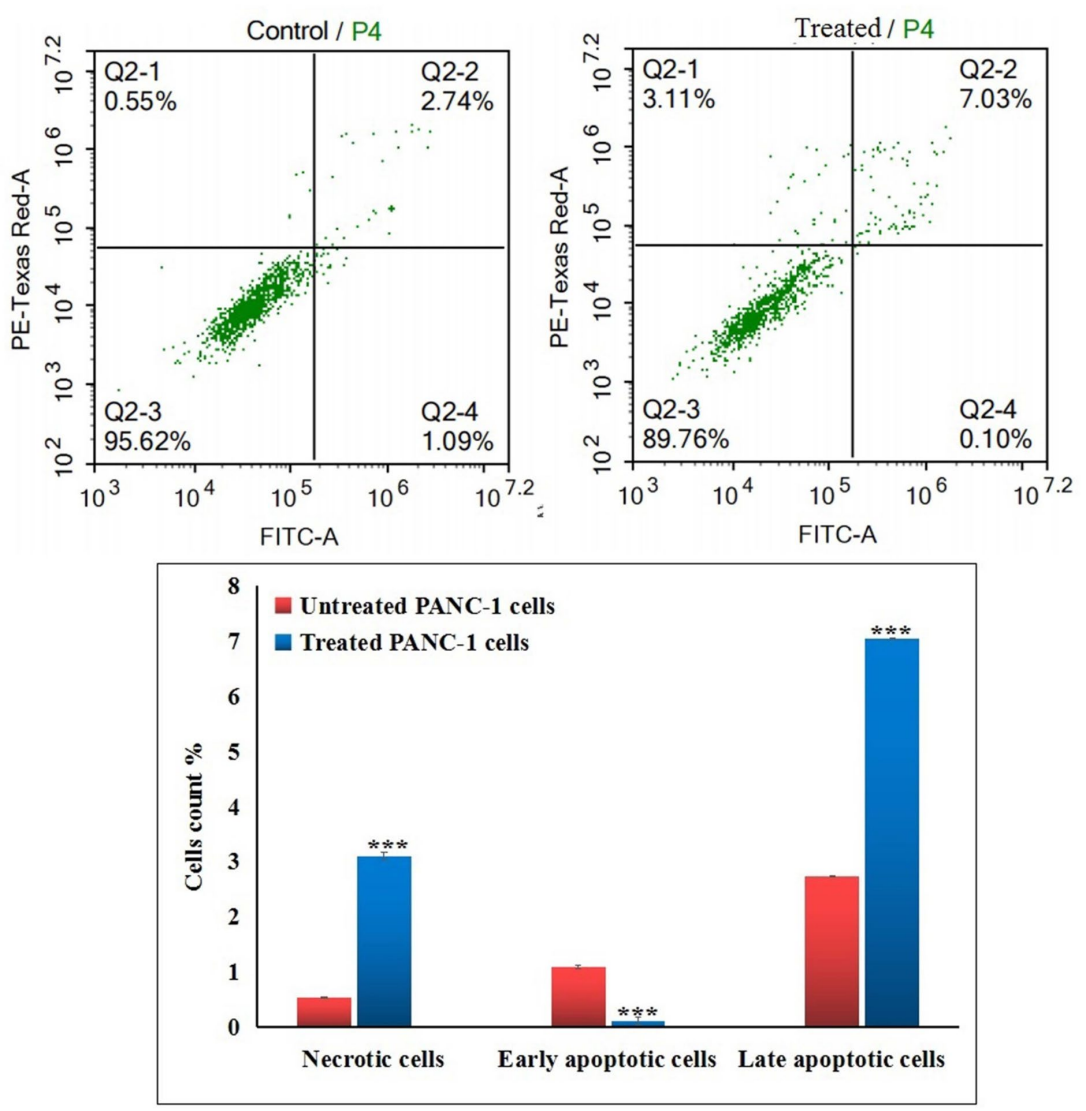
Apoptosis and necrosis induction in the untreated and treated pancreatic PANC-1 cancer cells with the IC50 concentration (152.40 µg/ml) of Ca(OH)_2_ NPs for 72 h. Q2-1 denotes necrosis phase; Q2-2 denotes late apoptosis phase, Q2-3 denotes normal viable cells and Q2-4 denotes early apoptosis phase.

Dual-channel Flow Cytometry analysis of PANC-1 cells revealed that exposure to Ca(OH)_2_ NPs at the IC50 concentration (152.40 µg/mL) forced PANC-1 cells into apoptotic and necrotic cell death (Fig. 6). This effect was manifested by a remarkable increase (*p* < 0.001) in the number of Ca(OH)_2_ NPs-treated PANC-1 cells in the late apoptotic and necrotic phases compared to the corresponding numbers in the untreated control PANC-1 cells (Fig. 6).

### Ca(OH)_2_ NPs highly disrupt p53, ND3 and Bcl2 expression in PANC-1 cells

The results of qRT-PCR demonstrated dramatic dysregulation of apoptotic p53, mitochondrial ND3 and anti-apoptotic Bcl2 gene expression in PANC-1 cells following treatment with Ca(OH)₂ NPs. As shown in Table 3, exposure to Ca(OH)₂ NPs at the IC50 concentration (152.40 µg/mL) for 72 h led to a significant upregulation of p53 and ND3 gene expression, accompanied by a marked downregulation of Bcl-2 gene expression compared to their expression level in the untreated control cells.

**Table 3:** The expression level of apoptotic p53, mitochondrial ND3 and anti-apoptotic Bcl2 genes in human pancreatic cancer (PANC-1) cells following exposure to Ca(OH)_2_ NPs at the IC50 concentration (152.40 µg/ml) for 72 h.

Cells	Treatment (µg/ml)	p53	ND3	Bcl2
PANC-1 cells	Ca(OH)_2_ NPs (0.00)	1.00 ± 0.00	1.00 ± 0.00	1.00 ± 0.00
	Ca(OH)_2_ NPs (152.40)	11.07 ± 1.76^**^	2.38 ± 0.12^***^	0.58 ± 0.07^**^

Results are expressed as mean ± SD.

**, ***: Indicates statistical significant difference from the compared untreated control cells at *p* < 0.01 and *p* < 0.001, respectively, using *independent student t-test*.

## Discussion

Pancreatic cancer remains one of the most aggressive malignancies with a poor prognosis and limited therapeutic options. The current standard of care, primarily chemotherapy, exhibits limited efficacy due to rapid tumor progression, drug resistance, and a dense stromal microenvironment that restricts drug penetration. Therefore, there is a critical need for novel therapeutic strategies that enhance treatment effectiveness while minimizing adverse effects. Among promising nanomaterials, Ca(OH)₂ NPs have attracted attention due to their unique physicochemical properties, including biocompatibility and a highly alkaline nature. These properties enable them to disrupt the acidic tumor microenvironment, potentially impairing cancer cell survival, inducing apoptosis, and inhibiting proliferation^11,22^. However, the therapeutic potential of Ca(OH)₂ NPs on pancreatic cancer remains largely unexplored. Therefore, the current study aims to estimate the effect of Ca(OH)₂ NPs on the viability of human normal OEC and pancreatic cancer PANC-1 cells. Additionally, the effect of Ca(OH)₂ NPs on genomic DNA integrity, ROS generation, mitochondrial membrane integrity and apoptosis induction in PANC-1 cells was investigated.

The findings of SRB cytotoxicity assay demonstrated a selective, concentration-dependent decrease in pancreatic cancer PANC-1 cell viability following treatment with Ca(OH)₂ NPs at five concentrations (0.1, 1, 10, 100, and 1000 µg/mL). This effect was significantly more pronounced in PANC-1 cancer cells compared to normal OEC cells, as indicated by the minimal cytotoxicity observed in OECs at the same concentrations and the high selectivity index of 3.16, suggesting a differential cytotoxic response. These results align with the potent, concentration-dependent cytotoxicity of Ca(OH)₂ NPs reported in hepatic Hep-G2 cancer cells in the recent study by Mohamed et al.^11^. However, our findings contrast with Mohamed et al.^11^, who observed a strong cytotoxic effect of Ca(OH)₂ NPs on normal human skin fibroblasts. This discrepancy may be attributed to differences in cell type susceptibility, nanoparticle concentration, or experimental conditions^23,24^.

The selective cytotoxicity of Ca(OH)₂ NPs demonstrated in this study may be attributed to the altered redox balance and metabolic state of cancer cells, which are more susceptible to oxidative stress compared to normal cells. The heightened ROS production induced by Ca(OH)₂ NPs plays a crucial role in selectively triggering cell death in cancer cells while sparing normal cells to a greater extent. Our data revealed a marked increase in intracellular ROS level in PANC-1 cancer cells following exposure to Ca(OH)₂ NPs at the IC50 concentration through a marked elevation in fluorescence intensity in Ca(OH)₂ NPs-treated PANC-1 cells compared to untreated cells. These results are consistent with recent findings by Mohamed et al.^11^. , which reported excessive ROS production induced by Ca(OH)₂ NPs in hepatic Hep-G2 cancer cells.

ROS serve a dual role in cellular physiology, functioning as crucial signaling molecules at low concentrations while becoming highly destructive when excessively generated Persistent ROS accumulation leads to genomic instability and mitochondrial dysfunction, ultimately triggering apoptosis through the intrinsic (mitochondrial) pathway. This ROS-driven process is particularly relevant in cancer, where oxidative stress influences cell fate, tipping the balance between survival and programmed cell death^25,26^.

Genomic instability, a defining characteristic of cancer and degenerative diseases, arises when excessive ROS overwhelm cellular antioxidant defenses, resulting in nuclear and mitochondrial DNA damage. The primary mechanisms include single- and double-strand DNA breaks, oxidative DNA damage, DNA repair pathways disruption and activation of tumor suppressor genes^27,28^. In this study, the loss of genomic stability in PANC-1 cells following Ca(OH)_2_ NPs exposure was evidenced by significant elevations in Comet assay parameters, indicating extensive DNA damage. This effect can be directly attributed to the demonstrated excessive ROS generation induced by Ca(OH)_2_ NPs in PANC-1 cells. These findings align with the recent study by Mohamed et al.^11^. , which also reported the Ca(OH)_2_ NPs-induced genomic instability in Hep-G2 hepatic cancer cells, further reinforcing the role of oxidative stress in DNA damage and genomic instability.

Mitochondria are both a major source and a primary target of ROS. Due to their high vulnerability to oxidative damage, excessive ROS can impair mitochondrial function and disrupt mitochondrial membrane permeability, leading to loss of mitochondrial membrane integrity, mitochondrial depolarization, cellular dysfunction and ultimately apoptosis^29–31^. In this study, the significant reduction in fluorescence intensity observed in Ca(OH)_2_ NPs-treated PANC-1 cells indicates a dramatic loss of mitochondrial membrane integrity, which can be directly attributed to the excessive ROS generation induced by Ca(OH)_2_ NPs in PANC-1 cells.

The observed reduction in mitochondrial membrane potential after 72 h of Ca(OH)_2_NPs exposure in PANC-1 cells demonstrates mitochondrial dysfunction, a known hallmark of apoptosis. However, attributing this effect solely to the direct action of the nanoparticles may overstate their role. Mitochondrial depolarization can also result from upstream apoptotic events such as oxidative stress, DNA damage, and caspase activation, all of which contribute to mitochondrial destabilization^32,33^. In our study, the loss of mitochondrial membrane potential likely reflects both the effects of Ca(OH)_2_ NPs exposure and the apoptosis-related pathways they induce. As established in the literature, mitochondrial membrane potential loss is typically a downstream consequence of intrinsic apoptotic signaling and not necessarily indicative of a direct mitochondrial target^34,35^. Therefore, while Ca(OH)_2_ NPs may play a role in initiating mitochondrial dysfunction, potentially through ROS generation and genotoxic stress, the resulting depolarization is better interpreted as part of the broader apoptotic cascade rather than evidence of direct mitochondrial targeting.

Apoptosis induction by Ca(OH)_2_ NPs in this study was manifested by a significant increases in the number of late apoptotic and necrotic PANC-1 cells, as detected using dual-channel Flow Cytometry. This observed apoptosis aligns with mitochondrial-dependent cell death mechanisms. Further supporting this, qRT-PCR analysis revealed a marked upregulation of the apoptotic p53 gene and mitochondrial ND3 gene, alongside a significant downregulation of the anti-apoptotic Bcl-2 gene in Ca(OH)_2_ NPs-treated PANC-1 cancer cells. Consistent with previous findings, p53 activation promotes apoptosis by directly inducing pro-apoptotic factors and indirectly suppressing anti-apoptotic Bcl2 gene expression^36,37^.

To further explore the mitochondrial involvement in Ca(OH)_2_ NPs-induced cytotoxicity, the expression level of the mitochondrial gene ND3 gene was assessed. ND3 gene encodes a subunit of mitochondrial respiratory chain Complex I and plays a critical role in maintaining mitochondrial bioenergetics and redox homeostasis. Although ND3 is not a classical marker of apoptosis, its dysregulation is associated with mitochondrial dysfunction, elevated ROS production, and the initiation of mitochondrial-mediated apoptosis. Emerging evidence suggests that ND3 and other mitochondrial genes contribute to cancer progression and apoptotic resistance; with ND3 functioning as an apoptosis-inducing factor by facilitating the release of pro-apoptotic molecules^38,39^. Therefore, assessing ND3 expression provides important mechanistic insight into mitochondrial disruptions caused by Ca(OH)2 NP exposure in PANC-1 cells. In this context, the demonstrated marked upregulation of p53 and ND3 coupled with the significant downregulation of anti-apoptotic Bcl2 gene drove PANC-1 cells toward mitochondrial-mediated apoptotic cell death.

The present study consequently provides the first evidence that Ca(OH)₂ NPs exert potent, selective cytotoxic and pro-apoptotic effects against human pancreatic PANC-1 cancer cells, while exhibiting minimal toxicity toward normal oral OECs epithelial cells. These findings suggest that Ca(OH)₂ NPs may serve as a promising preliminary candidate for further exploration as an anticancer agent. The observed effects appear to be mediated through excessive reactive oxygen species (ROS) generation, genomic DNA damage, and mitochondrial dysfunction leading to apoptosis. However, it is important to note that the current study is limited to in vitro experiments, and the findings should be interpreted with caution when extrapolating to in vivo or clinical contexts. Factors such as nanoparticle biodistribution, pharmacokinetics, systemic toxicity, and tumor microenvironment interactions remain to be thoroughly investigated. Therefore, while the results are encouraging, the therapeutic potential of Ca(OH)₂ NPs remains preliminary and requires comprehensive in vivo studies and mechanistic analyses to confirm their safety and efficacy in pancreatic cancer treatment.

## Conclusion

Collectively, this study demonstrated the potent differential cytotoxicity of Ca(OH)_2_ NPs in human pancreatic PANC-1 cancer cells, primarily mediated through ROS-driven genomic instability and mitochondrial apoptosis. The selective cytotoxic effect of Ca(OH)_2_ NPs was evidenced by a concentration-dependent reduction in PANC-1 cell viability, while only minimal toxicity was observed in normal OEC cells, highlighting their potential as a selective anticancer agent. Mechanistically, Ca(OH)_2_ NPs exposure led to excessive ROS generation, which induced significant DNA damage, as confirmed by elevated Comet assay parameters. The ROS-mediated mitochondrial dysfunction was further supported by a significant loss of mitochondrial membrane integrity, leading to apoptosis. qRT-PCR analysis revealed a marked upregulation of pro-apoptotic genes (p53 and ND3) and a downregulation of the anti-apoptotic Bcl-2 gene, confirming that Ca(OH)_2_ NPs trigger mitochondrial-mediated apoptotic cell death. The observed selective cytotoxicity against cancer cells while sparing normal cells suggests that Ca(OH)_2_ NPs hold promise as a potential therapeutic agent for pancreatic cancer treatment. However, further in vivo studies and mechanistic investigations are necessary to validate their clinical applicability and safety profile.

## Data Availability

Not applicable: this manuscript does not report data generation or analysis.The datasets used and/or analyzed during the current study are available from the corresponding author on reasonable request.
